# Draft Genome Sequences of Salmonella enterica subsp. *diarizonae* Serotype IIIb_61:I,v:1,5,(7) Strains Isolated from Wheat Grains

**DOI:** 10.1128/MRA.00035-21

**Published:** 2021-05-20

**Authors:** Pragathi B. Shridhar, Raghavendra G. Amachawadi, Mori Atobatele, Xiaorong Shi, Paige A. Adams, Randall K. Phebus, Tiruvoor G. Nagaraja

**Affiliations:** a Department of Diagnostic Medicine and Pathobiology, Kansas State University, Olathe, Kansas, USA; b Department of Clinical Sciences, Kansas State University, Manhattan, Kansas, USA; c Department of Applied Interdisciplinary Studies, Kansas State University, Manhattan, Kansas, USA; d Department of Animal Sciences and Industry, Kansas State University, Manhattan, Kansas, USA; University of Maryland School of Medicine

## Abstract

Salmonella enterica subsp. *diarizonae* serotypes are primarily involved in reptile-associated salmonellosis in humans. Here, we report the draft genome sequences of three *S. enterica* subsp. *diarizonae* strains belonging to the serotype IIIb_61:1,v:1,5,(7), isolated from wheat grains collected at the time of harvest. Strains of serotype IIIb_61:1,v:1,5,(7) have been isolated from feces of reptiles, cattle, and sheep and from infections in humans.

## Abstract (precis)

Salmonella enterica subsp. *diarizonae* serotypes are primarily involved in reptile-associated salmonellosis in humans. Here, we report the draft genome sequences of three subsp. *diarizonae* strains belonging to the serotype 61:1,v:1,5,(7), isolated from wheat grains collected at the time of harvest. Strains of serotype of 61:1,v:1,5,(7) have been isolated from feces of reptiles, cattle, and sheep and from infections in humans.

## ANNOUNCEMENT

Wheat grains are a raw commodity and can be contaminated during preharvest, harvest, transportation, and storage and at milling (1–3). The interest in the microbiological safety of wheat grains is because foodborne illness outbreaks linked to wheat flour have been reported (2). Salmonella illnesses linked to wheat flour have been reported in the United States and other countries (4–6).

Here, we report the draft genome sequences of three Salmonella enterica subsp. *diarizonae* strains isolated from wheat grains. The wheat grains were collected at harvest time from several states in the United States. First, 25 g of wheat grains were suspended in 225 ml of modified buffered peptone with pyruvate (mBPWp; Neogen Corp., Lansing, MI) and incubated at 37°C for 30 min. The suspension was then mashed in a Stomacher device (Seward 400, UK) and BagMixer 400 (Interscience, France) for 60 s. The wheat grain samples in the filter bags were incubated at 37°C for 5 h, after which novobiocin was added at the final concentration of 22 μg/ml. The filter bags with wheat grain samples were incubated at 37°C for an additional 19 h. An aliquot of 10 ml was pipetted into 90 ml Rappaport-Vassiliadis broth and incubated at 42°C for 24 h. A boiled lysate supernatant of the enriched suspension was subjected to a GeneClean Turbo kit (MP Biomedical, Solon, OH) and analyzed by real-time PCR targeting the *invA* and *pagC* genes (7). Samples positive for both genes were streaked onto Hektoen enteric (HE) agar and incubated at 37°C overnight. Three isolates were identified as Salmonella enterica subsp. *diarizonae* and were serotyped as IIIb_61:1,v:1,5,(7) by the National Veterinary Services Laboratories (7).

The strains were grown overnight in Difco Mueller-Hinton broth (Becton, Dickinson and Company, Sparks, MD) at 37°C. Genomic DNA was extracted using the ZymoBIOMICS DNA/RNA miniprep kit (Zymo Research, Irvine, CA) according to the manufacturer’s protocol. Isolated DNA was quantified using a Qubit device (Thermo Fisher Scientific, Waltham, MA). DNA libraries were prepared using the IonXpress Plus fragment library kit (Thermo Fisher Scientific) according to the manufacturer’s protocol. Library quantity was assessed with a Qubit device. The DNA libraries were subjected to whole-genome sequencing using the Thermo Fisher Ion S5 200-bp sequencing platform, generating single-end reads. The adapters were removed automatically from the reads using the IonTorrent software from the sequencer. Raw single-end reads were trimmed and processed using BBDuk (BBMap version 36.49 [8]) with read quality trimming parameters of qtrim = rl, trimq = 20, and minlen = 36. The trimmed FASTQ reads were assembled using SPAdes version 3.12 (9) with the –careful parameter. The assembled contigs were then processed through the CosmosID core genome single nucleotide polymorphism (SNP) typing pipeline to evaluate the phylogenetic placement and SNP differences for meaningful epidemiological inferences. Annotations were carried out using the NCBI Prokaryotic Genome Annotation Pipeline.

The numbers of reads generated for the three strains, 2018-3-62, 2018-3-200, and 2018-3-539, were 6,035,151, 7,292,824, and 6,341,864, respectively. The read lengths of the strains were 357 bp/read for 2018-3-62, 351 bp/read for 2018-3-200, and 351 bp/read 2018-3-539. Average read lengths of strains 2018-3-62, 2018-3-200, and 2018-3-539 were 188.23, 184.38, and 175.11 bp/read, respectively.

Antimicrobial resistance genes and the total number of phages were determined using ResFinder version 3.1 (https://cge.cbs.dtu.dk/services/ResFinder/) (10) and Phage Search Tool Enhanced Release (PHASTER; http://phaster.ca/) (11, 12), respectively, using default parameters. All three strains carried Salmonella phage SEN22 and genes encoding resistance for aminoglycoside antibiotics [*aac(6′)-laa*]. The three strains belonged to the sequence type 243 (ST-243), as determined *in silico* with the MLST 2.0 (https://cge.cbs.dtu.dk/services/MLST/) tool using default parameters (13, 14).

Salmonella enterica subsp. *diarizonae* is most commonly found in cold-blooded animals, particularly snakes (15, 16), and IIIb_61:I,v:1,5,(7) is one of the main serotypes involved in reptile-associated salmonellosis in humans (15, 17–19).

### Data availability.

The whole-genome sequences of these three strains of serotype IIIb_61:1,v:1,5,(7) have been deposited in DDBJ/ENA/GenBank under the accession numbers JAEKIU010000000, JAEILY010000000, and JAEILX010000000 (see Table 1). The raw reads have been submitted to the NCBI SRA under accession number PRJNA684586. The versions described in this paper are the first draft genome sequences for S. enterica subsp. *diarizonae* strains available in GenBank.

**TABLE 1:** Genome characteristics of Salmonella enterica subsp. *diarizonae* strains isolated from wheat grains

Strain	Source of wheat grains	Serotype	Genome size (Mb)	No. of contigs	*N*_50_ (bp)	GC content (%)	GenBank accession no.
2018-3-62	South Dakota	IIIb_61:I,v:1,5,(7)	4.7	54	207,462	51.5	JAEKIU010000001–JAEKIU010000054
2018-3-200	Missouri	IIIb_61:I,v:1,5,(7)	4.7	52	265,526	51.5	JAEILY010000001–JAEILY010000052
2018-3-539	Maryland	IIIb_61:I,v:1,5,(7)	4.7	56	187,569	51.5	JAEILX010000001–JAEILX010000056
